# A Chemical Risk Ranking and Scoring Method for the Selection of Harmful Substances to be Specially Controlled in Occupational Environments

**DOI:** 10.3390/ijerph111112001

**Published:** 2014-11-20

**Authors:** Saemi Shin, Hyung-Il Moon, Kwon Seob Lee, Mun Ki Hong, Sang-Hoon Byeon

**Affiliations:** 1 Department of Environmental Health, College of Health Science, Korea University, Seoul 136-703, Korea; E-Mails: saemishin@naver.com (S.S.); himoon86@korea.ac.kr (H.-I.M.); 2 Occupational Safety & Health Research Institute, Korea Occupational Safety & Health Agency, Ulsan 339-30, Korea; E-Mails: iks0620@hanmail.net (K.S.L.); hongmnk@hanmail.net (M.K.H.)

**Keywords:** risk ranking, scoring method, occupational environment, chemical management

## Abstract

This study aimed to devise a method for prioritizing hazardous chemicals for further regulatory action. To accomplish this objective, we chose appropriate indicators and algorithms. Nine indicators from the Globally Harmonized System of Classification and Labeling of Chemicals were used to identify categories to which the authors assigned numerical scores. Exposure indicators included handling volume, distribution, and exposure level. To test the method devised by this study, sixty-two harmful substances controlled by the Occupational Safety and Health Act in Korea, including acrylamide, acrylonitrile, and styrene were ranked using this proposed method. The correlation coefficients between total score and each indicator ranged from 0.160 to 0.641, and those between total score and hazard indicators ranged from 0.603 to 0.641. The latter were higher than the correlation coefficients between total score and exposure indicators, which ranged from 0.160 to 0.421. Correlations between individual indicators were low (−0.240 to 0.376), except for those between handling volume and distribution (0.613), suggesting that each indicator was not strongly correlated. The low correlations between each indicator mean that the indicators and independent and were well chosen for prioritizing harmful chemicals. This method proposed by this study can improve the cost efficiency of chemical management as utilized in occupational regulatory systems.

## 1. Introduction

A wide variety of harmful chemicals, both as raw materials and byproducts, exist in industrial workplaces [1], and the number of such chemicals has been steadily increasing since the Industrial Revolution. Each year, about 200 to 300 new chemicals are introduced into the domestic market for industrial use [2], and there are presently about 45,000 types of chemicals used in Korean workplaces. However, with the increasing use of harmful chemicals, there has been an accompanying increase in the incidence of occupational disease. In 2010, there was an 18.2% increase in deaths, and a 5.5% increase in injuries related to hazardous chemical exposure as compared to 2009 [2].

South Korea presently manages 108 substances of which the manufacture, import, transfer, offer, or usage are prohibited, 13 substances of which the manufacture, import, transfer, offer, or usage are permitted, 13 harmful agents for which permissible exposure limits are required, 167 harmful substances to be controlled, 190 harmful substances subject to monitoring in the work environment, and 649 harmful agents for which occupational exposure limits are required.

The current Korea Occupational Safety and Health Act (OSH Act) for industrial accident prevention was issued in 1981, but the establishment of occupational exposure limits has been insufficient because many chemicals are imported from foreign countries such as Japan or the USA. Thus, since 2012, the Ministry of Employment and Labor (MOEL) in South Korea has managed chemicals by establishing and revising threshold limit values and continuously selecting specially managed chemicals [2]. The MOEL designated 167 substances as “harmful substances to be controlled” in 2002, in which are included harmful agents for which occupational exposure limits and additional control are required, and identified 9 substances as “carcinogens” that had a critical toxicity, including carcinogenicity, in harmful substances to be controlled in 2010. According to amendments made to the OSH Act in 2012, the term “carcinogen” was changed to “harmful substances to be specially controlled”, reflecting the comprehensive risk derived from such chemicals.

The MOEL has since decided to expand the scale of substances to be specially controlled. For the selection of additional harmful substances to be specially controlled, a standard was needed. While it is essential to determine all routes of potential harm associated with a given chemical and assign an accurate threshold limit, the time and resources necessary to accomplish this task are lacking. In this respect, the proposal of a priority screening method for specially managed chemicals would represent an important starting point [3,4,5,6].

The chemical ranking and scoring (CRS) method, which categorizes chemicals based on their toxicity exposure effects, has been adopted by many countries over the past 20 years [7,8,9,10,11,12,13]. Park *et al.* (2005) proposed a Korean chemical priority ranking method (CRS-Korea) for selecting high-priority chemicals that considers a given chemical’s health and ecological effects. However, since this method was developed to assign priority to chemicals potentially harmful to the ecological environment, it is not suitable for an occupational environment. Several pilot studies within the Korean industrial health field have assessed priority-screening methods [6,14], but these do not serve the purposes of the MOEL. Two preceding studies were based on the human health aspect of the European Union Risk Ranking Method (EURAM). EURAM establishes a hazard score for an assigned weighted value of the most prior health effect, while implementing an exposure score that is proportional to log-scaled emissions. The total score was mostly related to the exposure score. For the screening of highly toxic chemicals that may potentially exert irreversible effects on human health, the MOEL wanted to make the method relatively hazard-driven for previous studies.

The MOEL is making an effort to increase the number of harmful substances to be specially controlled (about 16 in 2013), in order to reduce the incidence of occupational diseases. Article 24 of the Occupational Safety and Health Act specifies the management of chemicals with the potential to damage health, such as raw materials in organic compounds, metals, acids, alkaline chemicals, and gaseous chemicals [15]. In addition, harmful substances to be specially controlled include those with the potential for carcinogenicity, germ cell mutagenicity, and reproductive toxicity (hereafter, CMR). Thus, workplaces in which harmful substances to be specially controlled are used should have enclosure equipment or a local exhaust ventilation system. Moreover, workers should be informed, preferably in writing, of the potential harm associated with handing such materials [16].

The hazard characterization of a given chemical should be the primary factor in the MOEL categorization. Thus, the aim of the present study was to develop an appropriate method for selecting harmful substances to be specially controlled in occupational environments.

## 2. Methods

### 2.1. Chemical Risk Ranking and Scoring (CRIRS) Method

The human health section of EURAM [1] was partially revised to develop a method suitable for the purposes of the present study. We used this section because while other ranking and scoring methods encompassed both environmental and human health, we wanted to focus solely on human health. We first compared the hazard and exposure indicators, and scoring methods of EURAM and CRIRS as in Table 1.

**Figure 1:**
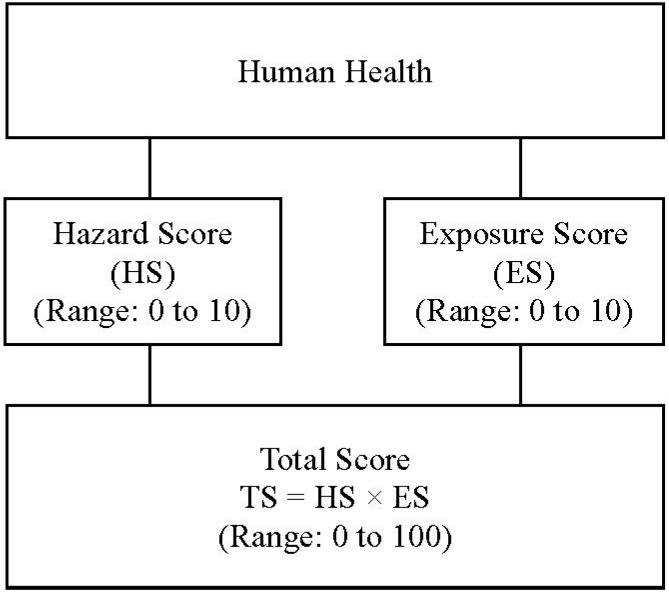
Algorithm for the priority ranking of workplace chemicals.

Total scores for each chemical were calculated by multiplying the hazard score by the exposure score, as shown in Figure 1. The hazard indicator scores used to calculate the hazard score ranged from 0 to 100 points, and the exposure indicator scores used to calculate the exposure score also ranged from 0 to 100 points. A maximum of 10 points for each score were normalized, and the total scores were calculated by multiplying the hazard score by the exposure score [1].

**Table 1:** Indicators and scoring methods of European Union Risk Ranking Method (EURAM) and Chemical Risk Ranking and Scoring (CRIRS).

Category	Euram	Crirs
Hazard indicators	Carcinogenicity Genetic toxicity Reproductive toxicity Respiratory sensitization Repeated dose toxicity Acute toxicity Irritation Skin sensitization	Acute toxicity (oral, dermal)
		Acute toxicity (oral, dermal)
		Irritation (skin, eyes)
		Sensitization (skin, respiratory)
		Aspiration hazard
		Germ cell mutagenicity
		Reproductive toxicity
		Carcinogenicity
		Target organ toxicity (repeated)
Exposure indicator	Emission Distribution	Handling volume
		The number of Workers
		The number of workplaces
		Volatility/Dustiness
		Measured exposure
Scoring method	Multiplying hazard and exposure scores that are normalized up to 10	Multiplying hazard and exposure scores that are normalized up to 10

The normalized hazard/exposure indicator value (hv_i_) for each raw hazard/exposure indicator value (HV_i_) in the “i”th row was calculated as follows:

(1) $$\text{Normalized}\;\text{value}\;(\text{h}v_{i})\;=\;\frac{HV_{i\;}-\;\;HV_{\text{min}}}{HV_{\text{max}}\;-\;HV_{\text{min}}}\times 10$$

HV_i_: the hazard/exposure indicator value of indicator I;
HV_min_: the minimum value for HV_i_;
HV_max_: the maximum value for HV_i__._

### 2.2. Hazard Indicator Scoring

The hazard classification criteria of the “Risk Classification Guidelines of Chemicals (KOSHA GUIDE W-16-2012)” [17] of the Korea Occupational Safety and Health Agency (KOSHA) were modified for the purposes of the present study, and hazard scores were derived for each of these. Hazard scores for each chemical consisted of the sums of the weighted scores for CMR, and other toxicity and health-hazard classifications (particularly those from the Globally Harmonized System of Classification and Labeling of Chemicals (GHS)). With regard to CMR classification, we used information from the European Union (EU) [18], International Agency for Research on Cancer (IARC) [19], National Toxicology Program (NTP) [20], American Conference of Governmental Industrial Hygienists (ACGIH) [21], and Occupational Safety and Health Administration (OSHA) [22] databases, all of which are included in the source database in Korea for GHS classification, and the most conservative values were used. Similarly, the GHS classifications of the National Institute of Technology and Evaluation (NITE), the Ministry of Environment (MOE), and the National Emergency Management Agency (NEMA) were incorporated into the GHS classifications used by this study [23]. While we clearly delineated the various hazards associated with a given chemical, each hazard was factored into the total score.

According to the classification criteria for hazard classes in the “Risk Assessment Guidelines of Chemicals (KOSHA GUIDE W-6-2012)” [24], if a chemical has multiple routes of exposure (e.g., oral, skin, inhalation exposure) the associated hazard score can exceed those of chemicals with CMR. As shown in Table 2, CMR toxicity was assigned a higher weight than those of other forms of toxicity, and acute toxicity, irritation, and sensitization were all adjusted to comprise a single indicator, regardless of exposure route. However, acute toxicity from inhalation was designated as a separate item, given its potential as a major occupational hazard. In addition, because estimations regarding target organ toxicity (single exposure) were based on experiments that utilized animals under conditions that were not typical of a workplace environment, target organ toxicity (single exposure) was excluded as a priority selection indicator in this study. The overall scores were adjusted to account for the integration of some indicators. Using this method, a total score of 100 was set for nine hazard indicators.

**Table 2:** Scoring for the classification of hazard indicators.

Term	Hazard Characteristics	Scoring by Hazard Class							
		Class 1		Class 2	Class 3	Class 4	No Data	Not Classified	Not Applicable
		1A	1B						
T1	Acute toxicity (oral, dermal)	6		4	2	1	6	0	0
T2	Acute toxicity (inhalation)	5		3	2	1	5	0	0
T3	Irritation (skin, eyes)	6		4	-	-	6	0	0
T4	Sensitization (skin, respiratory)	9		-	-	-	9	0	0
T5	Aspiration hazard	5		3	-	-	5	0	0
T6	Germ cell mutagenicity	20	16	12	-	-	20	0	0
T7	Reproductive toxicity	20	16	12	-	-	20	0	0
T8	Carcinogenicity	20	16	12	-	-	20	0	0
T9	Target organ toxicity (repeated)	9		7	-	-	9	0	0

### 2.3. Exposure Indicator Scoring

The indicators related to direct or indirect chemical exposure were classified into degradation or transformation potential-relevant indicators, mobility- or partitioning-relevant indicators, estimated dose-, environmental occurrence-, concentration-, or release-relevant indicators, and exposure frequency or intensity indicators (receptor characteristics) [3]. In the field of occupational health, as exposure typically occurs in a confined space through the respiratory tract, the use of degradation- and transformation-relevant indicators, or mobility- or partitioning-relevant indicators, is less likely. However, the degree of atmospheric dustiness, volatility, and space should be considered [25]. Therefore, in this study, volume and emissions, distribution-related indicators, and measured exposure were summed to yield an overall exposure indicator. The indicators related to volume and emissions were circulation, handling volume, number of workers, and number of workplaces in which the chemical could be used. Based on Article 42 of the Occupational Health and Safety Act, the MOEL requires that employers assess the work environment once every six months. In addition, information on the total annual number of workers and workplaces is gathered by the “Survey of Exposure to Hazardous Substances”, conducted once every five years in compliance with the Enforcement Regulations of the Occupational Health and Safety Act. Information on the circulation of chemicals is also gathered by the “Circulation Research Chemicals,” conducted once every four years by the MOE in accordance with Article 9 of the Toxic Chemicals Control Act.

The data related to the circulation of a given chemical (provided by the MOE) includes not only its actual use in the workplace, but also the degree of its manufacture, import, and export, including its use. To identify the risk of a given chemical to workers, the total handling volume provided by the MOEL was deemed suitable. If the handling volume had not been identified, the amount used in circulation was an alternative source of information. A classification standard based on weight is ideal for chemical substance management, thus, the classification criteria in the present study were as follows: 100 kg is the hazard standard set by the MOEL and MOE; 1 ton is the registration standard set by REACH; 10 tons is the standard volume set by REACH that requires chemical safety assessment; and 1000 tons or more is the Organization for Economic Cooperation and Development (OECD) High Production Volume (HPV) standard. The score was based on the total handling volume, and the maximum score was set at 30 points.

The categories related to distribution were physical properties, and the number of exposed workers and workplaces. These three indicators were summed with equal weights when calculating the distribution score. Workers, consumers, and humans exposed indirectly via the environment were all considered by the EURAM. Of these three populations, the workers were of primary concern in the present study. The EURAM reflected this size of exposed populations, but only at a very basic level by considering the chemicals’ physicochemical properties, such as vapor pressure, boiling point, and log K_ow_, due to the difficulties associated with establishing a simple ranking method that accurately estimates population sizes [1]. Physicochemical properties have been also used as indicators in generic risk-ranking methods, such as the KOSHA GUIDE W-6-2012, Chemical Hazard Risk Management (CHARM) [26], and Control of Substances Hazardous to Health (COSHH) [27], which are essential for predicting exposure potentials. In this study, physical properties and the number of workers and workplaces were used as distribution indicators and equally assigned 10 points, thus yielding a total of 30 points.

To determine the number of exposed workers, we referred to the classification standard of the Occupational Safety and Health Act. As shown in Table 3, the criteria for classifying chemicals by the number of exposed workers and workplaces were as follows: Class 5, which was based on the incomplete application standards of the Labor Standards Act, was an exposure of less than 5 workers; Class 4, which was based on the overall application standards of the Labor Standards Act, was an exposure of between 5 and 49 workers; Class 3, based on the contracting-out standards of specialized institutions for health management, was an exposure of between 50 and 99 workers; Class 3, which was based on the organizational standards of the Occupational Safety and Health Committee, was an exposure of between 100 and 299 workers; and Class 2, which was based on the appointing standards of health managers, was an exposure of more than 300 workers. The workplace data had no prior example for a standard of assessment or management, so government agencies, experts, and the present authors reached an agreement that used the same standard as with exposed workers. Ten points were given to each indicator of exposed workers and workplaces.

**Table 3:** Classification criteria for exposure indicators.

Term	Exposure Indicator		Classification Criteria				
			Class 1	Class 2	Class 3	Class 4	Class 5
E1	Handling volume (ton/year)		>1000	10–1000	1–10	0.1–1	<0.1
	Circulation (ton/year)		>1000	10–1000	1–10	0.1–1	<0.1
E2	Worker numbers (No.)		>300	100–300	50–100	5–50	<5
E3	Workplace numbers (No.)		>300	100–300	50–100	5–50	<5
E4	Volatility/Dustiness	Liquid/Gas (boiling point, °C)	<50	50–150	>150	-	-
		Solid (as per ACGIH guidelines)	To have V, IFV, H, R	Powders or crystalline	Pellet-like, non-friable solids *etc.*	-	-
E5	Measured exposure	Liquid/Gas (ppm)	>500	50–500	5–50	0.5–5	<0.5
		Solid (mg/m^3^)	>10	1–10	0.1–1	0.01–0.1	

V: vapor and aerosol; IFV: Inhalable fraction and vapor; H: Aerosol only; R: Respirable fraction.

“Physical property” referred to the volatility and dustiness of a chemical, similar to the criteria set by CHARM and COSHH. However, in this generic risk-ranking method, we adopted a different volatility indicator. The exposure index in the KOSHA GUIDE (W-6-2012) was reflective of vapor pressure, while on the other hand, the exposure index in CHARM reflected boiling point. Volatile organic compounds were defined by their boiling points in “Indoor air quality: organic pollutants,” written by the World Health Organization [28], and in the “Directive 2004/42/CE of the European Parliament and the Council” [29] in the EU. In the present study, the logarithmic values of boiling point and vapor pressure were highly correlated (R = −0.95, *p* = 0.000). Thus, we thought it unnecessary to include both boiling point and vapor pressure values in the representation of a substance’s physical properties. Therefore, in this study, volatility was determined by boiling point. Volatility and dustiness each had three classes, which were based on the guidelines of the ACGIH [30] and CHARM.

“Harmful substances to be controlled” as defined by the OSH Act were legally measured on a regular basis, so the measured exposure can be used as an exposure indicator. These are graded by the predicted exposure ranges of the exposure bands of COSHH. As the measured exposure directly reflects the workplace environment and is considered to be the determining factor, the maximum score for this indicator was 40 points, which was higher than that of the other indicators. Exceeding each predicted exposure range resulted in 8 points being added to the chemical’s score, for a total of 40 points.

Handling volume was multiplied by distribution, as stated in the above paragraph. However, in addition to handling volume and distribution, the measured exposure was used as an indicator in this study. To ensure that no one factor dominated the algorithm, all indicators were added to derive an overall exposure value. Therefore, the 100-point exposure indicator reflected handling volume (30 points), number of workers (10 points), number of workplaces (10 points), physical properties (10 points), and exposure level (40 points) (Table 4).

**Table 4:** Scoring for classification of exposure indicators.

Term	Exposure Indicator		Scoring					
			Class 1	Class 2	Class 3	Class 4	Class 5	Not Classified
E1	Handling volume (replaced with circulation if data were missing)		30	24	18	12	6	0
E2	Worker numbers		10	8	6	4	2	0
E3	Workplace numbers		10	8	6	4	2	0
E4	Volatility/Dustiness	Liquid/Gas	10	8	6	-	-	0
		Solid	10	8	6	-	-	0
E5	Measured exposure		40	32	24	16	8	0

## 3. Results and Discussion

### 3.1. Evaluation of the Priority Setting

In this study, the CRIRS method was developed using existing chemical priority selection methods. These existing techniques allowed us to create a method to assess the degree of risk for each indicator (hazard/exposure). In this study, we ranked 62 harmful substances to be controlled, for the designation of harmful substances to be specially controlled. All 62 chemicals had CMR toxicity according to the Occupational Safety and Health Act, prescribing these harmful substances to be specially controlled should they be selected from amongst the CMR substances. Although exposure indicators (measured exposure and volume used domestically) were low for these CMR chemicals, examination of the regulation level intensification for such chemicals indicated that they had highly harmful properties, particularly in the case of carcinogens. From this perspective, the indicator scores for highly toxic chemicals could compare with those of low-toxicity but high-exposure chemicals.

The 10 highest-ranked chemicals (about 16%) of the 62 harmful substances to be controlled are presented in Table 5. Their average total score is 49.80, which is 12.3 times as large as the average total score of the 10 lowest ranked chemicals (4.05). The substance that had the highest hazard score had a high ranking, but the substance that had the highest exposure score was ranked 37 and had a relatively low ranking.

**Table 5:** Top 10 ranked chemicals used in the algorithm.

Chemicals	CAS No.	Hazard Score	Exposure Score	Total Score
Styrene	100-42-5	6.98	9.60	67.05
Acrylamide	79-06-1	8.89	6.80	60.44
Acrylonitrile	107-13-1	8.41	6.80	57.21
Cyclohexanone	108-94-1	6.35	7.60	48.25
Ethyleneimine	151-56-4	10.00	4.80	48.00
Stoddard solvents	8052-41-3	5.08	9.20	46.73
Dimethyl sulfate	77-78-1	6.98	6.40	44.70
Perchloroethylene	127-18-4	4.92	8.80	43.30
Dimethylformamide	68-12-2	4.60	9.20	42.35
Ethyl acrylate	140-88-3	5.56	7.20	40.00

In conventional chemical-ranking methods, particularly those concerning general ecological factors and human health, carcinogenic heavy metals that have high accumulation tendencies are typically ranked higher [10,31,32,33]. However, because the present study based priority ranking on the risk to humans in the workplace environment, the rankings naturally differed. In addition, because cadmium, nickel, chrome, and lead were already designated as specially managed chemicals, they were excluded from the analysis.

### 3.2. Correlation Analysis of Individual Indicators and Total Score

In the priority selection method, a regression analysis was performed to determine to what extent specific indicators affected the total score. Correlation coefficients for each indicator and total score are shown in Table 6. The correlation between CMR and total score was 0.641, while that between other toxicity indicators and total score was 0.603. As mentioned previously, the hazard indicator score was the sum of the CMR and other toxicity scores; the correlations of CMR and other toxicity indicators with the hazard indicator score were 0.911 and 0.725, respectively.

**Table 6:** Correlation coefficients (r) from a linear regression analysis of indicator scores and total scores.

Indicator	r	
	r_1_	r_2_
CMR	0.641 *	0.911 *
Other toxicity	0.603 *	0.725 *
Handling volume	0.421 *	0.855 *
Distribution	0.305 *	0.874 *
Measured exposure	0.160	0.580 *

r_1_: Correlation coefficients between indicator scores and total scores; r_2_: Correlation coefficients between indicator scores and hazard/exposure indicator scores; Hazard score: The normalized value for CMR value + other toxicity value; Exposure score: The normalized values for handling volume value + distribution value (the number of workers, the number of workplaces, volatility/dustiness) + measured exposure value; * *p* < 0.05.

The correlations between handling volume, distribution, and exposure level and total score were 0.421, 0.305, and 0.160, respectively. Total score and handling volume were significantly correlated; in contrast, distribution and exposure level were not significantly correlated with total score. Moreover, these correlations were weaker overall as compared to the toxicity correlations. This suggests that the hazard indicator may affect total score to a greater degree than the exposure indicator. Therefore, this result supports the purpose of this study. Further, the exposure indicator score reflects the total sum of the handling volume, distribution, and exposure level scores. The correlations of total handling volume and distribution with total score were high (0.911, and 0.725, respectively); however, that of exposure level was the lowest (0.580). In other words, exposure level did not provide a large contribution to the exposure indicator score. This may be due to the fact that exposure levels for most chemicals were less than 5 ppm or 0.1 mg/m^3^; thus, most chemicals could be classified as lower than Class 4.

**Figure 2:**
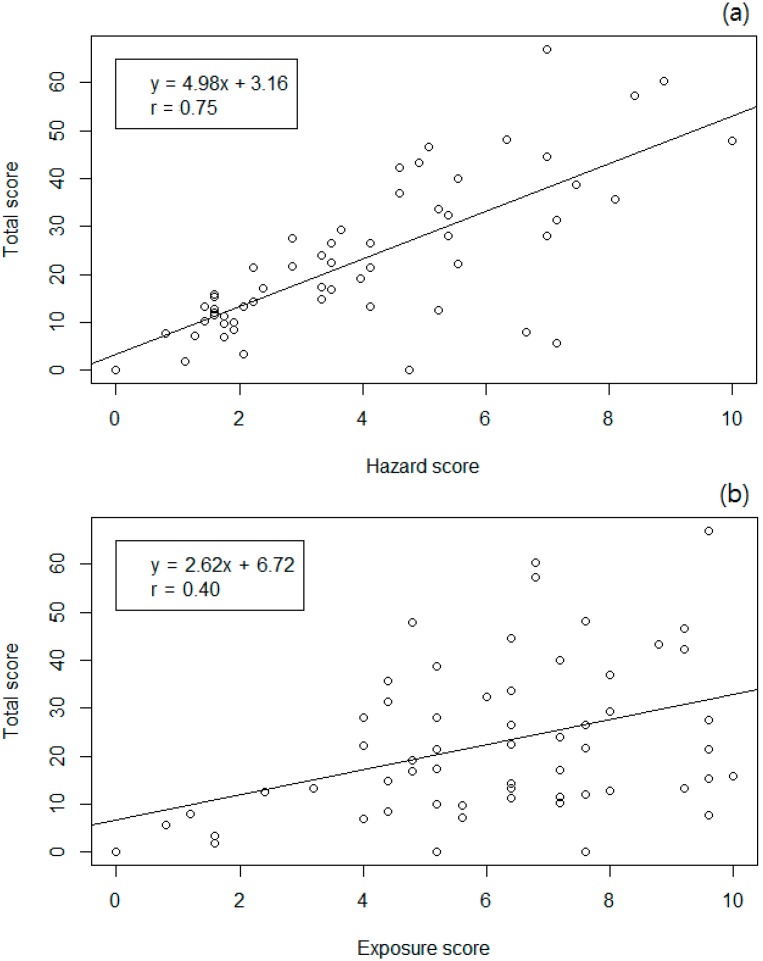

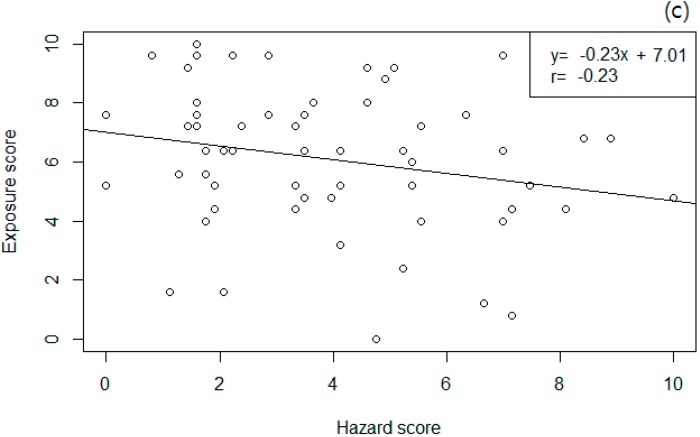
(**a**) The correlation between hazard score and total score; (**b**) The correlation between exposure score and total score; (**c**) The correlation between hazard score and exposure score.

In this study, CMR and other toxicities had the greatest impact on total score in the ranking of managed chemicals. As shown in Figure 2a,b, the correlation between hazard indicator and total score was 0.75, which was higher than that between exposure indicator and total score (0.40). As shown in Figure 2c the correlation between hazard and exposure score was −0.22; this weak correlation suggests that the exposure and hazard indicators are independent.

The results of the correlation analysis are shown in Table 7. The correlation between other toxicities and CMR, handling volume and distribution, and distribution and measured exposure was statistically significant. The correlation between distribution and handing volume had an especially high coefficient (0.613). Since distribution includes the sum of the workers and workplaces, its correlation with handling volume is reasonable. However, the r values for the other correlations were lower than 0.5, which suggests these two indicators are not strongly correlated. In addition, most correlations were not statistically significant, suggesting that the indicators were appropriate because of their independence.

**Table 7:** Correlation coefficients (r) from the linear regression analysis for individual indicator scores.

Term	CMR	Other Toxicity	Handling Volume	Distribution	Measured Exposure
CMR	1.000				
Other toxicity	0.375 *	1.000			
Handling volume	−0.204	0.035	1.000		
Distribution	−0.240	−0.135	0.613 *	1.000	
Measured exposure	−0.146	−0.140	0.256 *	0.355 *	1.000

Distribution value = the number of workers, the number of workplaces, volatility/dustiness; * *p* < 0.05.

## 4. Conclusions

This study was performed to develop a prioritization method to facilitate the special management of hazardous chemicals in the occupational environment. By applying this method, we prioritized 62 CMR chemicals, and found that the hazard indicator had a stronger correlation with the total score than did the exposure indicator. Furthermore, a weak correlation was observed between the exposure and hazard indicators, suggesting that the selected indicators were well chosen because of their independence. The CRIRS is a priority selection method that considers both hazard and exposure indicators; this method can improve the cost efficiency of chemical management as utilized in occupational environments.
